# Supplementary material for: Human whole-exome genotype data for Alzheimer’s disease
Source: Nat Commun. 2024 Jan 23;15:684. doi: 10.1038/s41467-024-44781-7 (PMC10805795; doi:10.1038/s41467-024-44781-7)
Supplement: Supplementary file 1 — Supplementary Information [file 41467_2024_44781_MOESM1_ESM.pdf]

Title: Human whole exome genotype data for Alzheimer's Disease

SUPPLEMENTARY FIGURES

Supplementary Fig. 1 – Comparison of 20x coverage at capture regions (x-axis) versus coding regions (exons) inside the capture regions (y-axis) for 100 samples. Color in the plot represents different sequencing centers (Seq.center) while the plot was is stratified by capture kits (Capture_exonic). Samples were randomly selected based on different studies-sequencing_center-capture combinations. The 20x coverage at the capture regions (88.5±7.8) is similar to that of the coding regions (88.2±2.5) across all samples (N = 20,504 samples in total), yet the 20x coverage values in the coding regions of the capture regions are more uniform across samples.

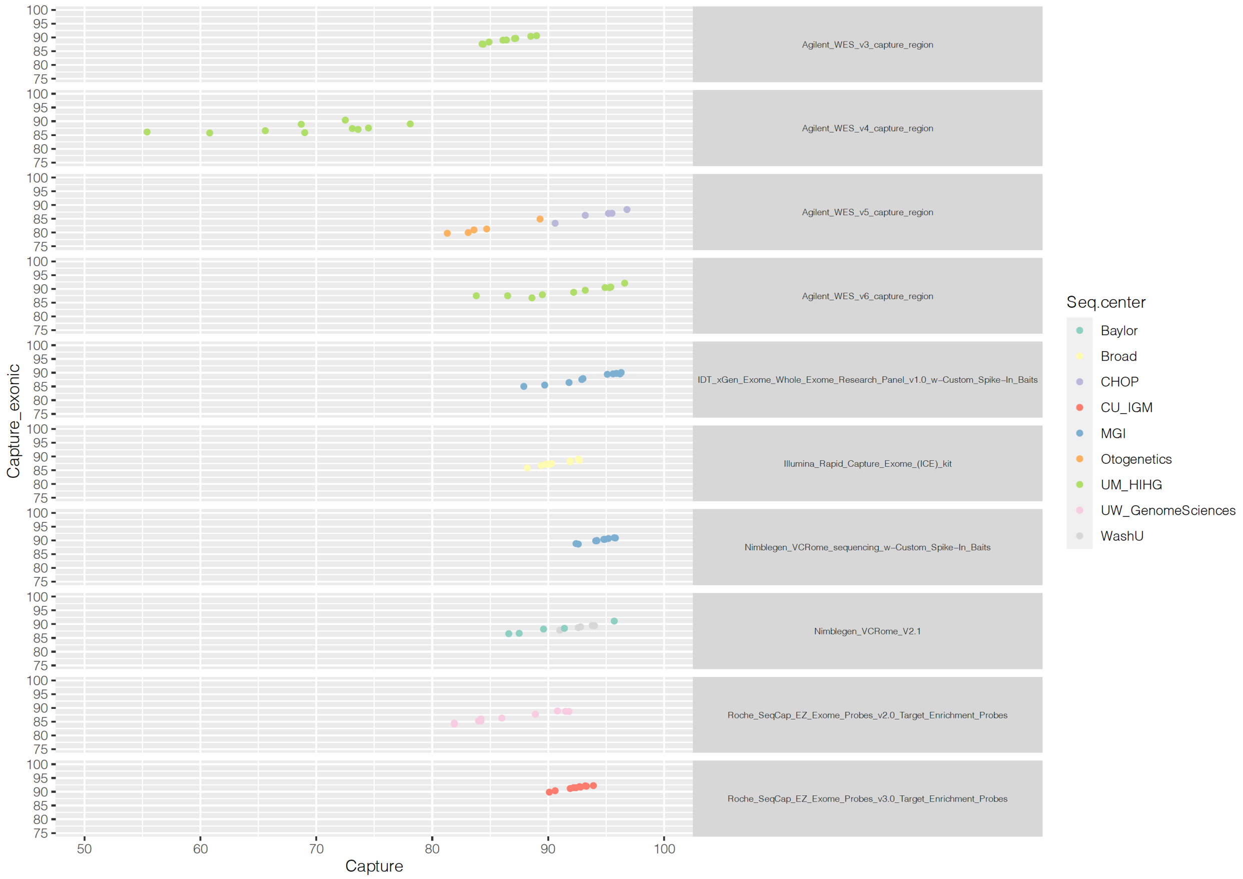

Supplementary Fig. 2 – Frequencies of synonymous variants between cases and controls by QC subsets. Selected QC subsets shown here are studies of biggest sample sizes. Each dot in the boxplot is a gene. N for the four groups (from top to bottom): 3861, 6072, 4585 and 3157 subjects. Quality metrics included i) Percentage of mapped reads, ii) Percentage of duplicated reads, iii) Percentage of paired reads, and iv) Quality of reads based on Q30 score. For each box plot, the centre line represents the median value, the minimum of the whisker represents the 1st quantile (25th percentile) and the maximum of the whisker represents the 3rd quantile (75th percentile).

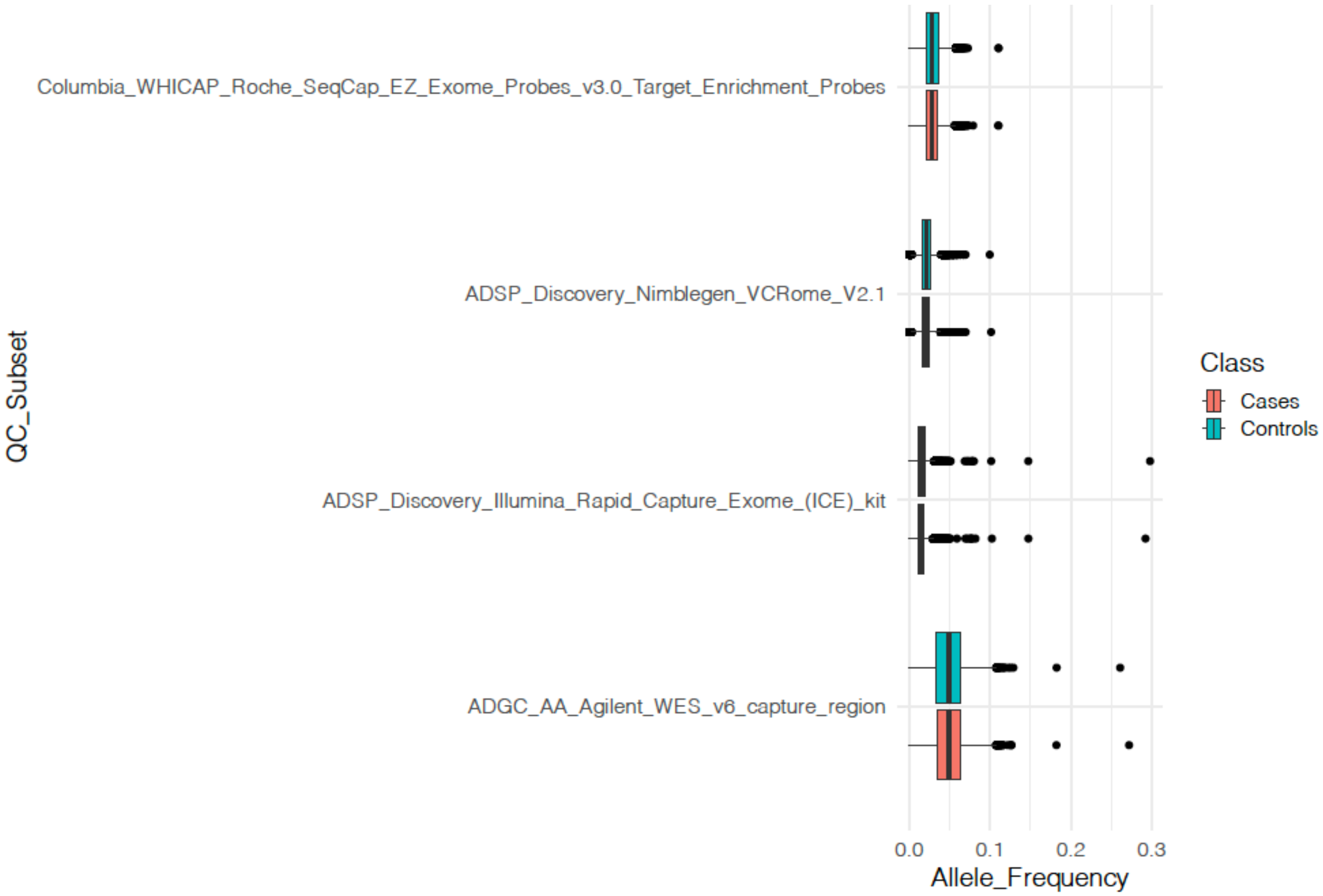

Supplementary Fig. 3 – The ratios of Post-QC to Pre-QC genotype counts (REF/REF, REF/ALT, and ALT/ALT) for each genotype per QC subset (exclude the three QC subsets with no controls).

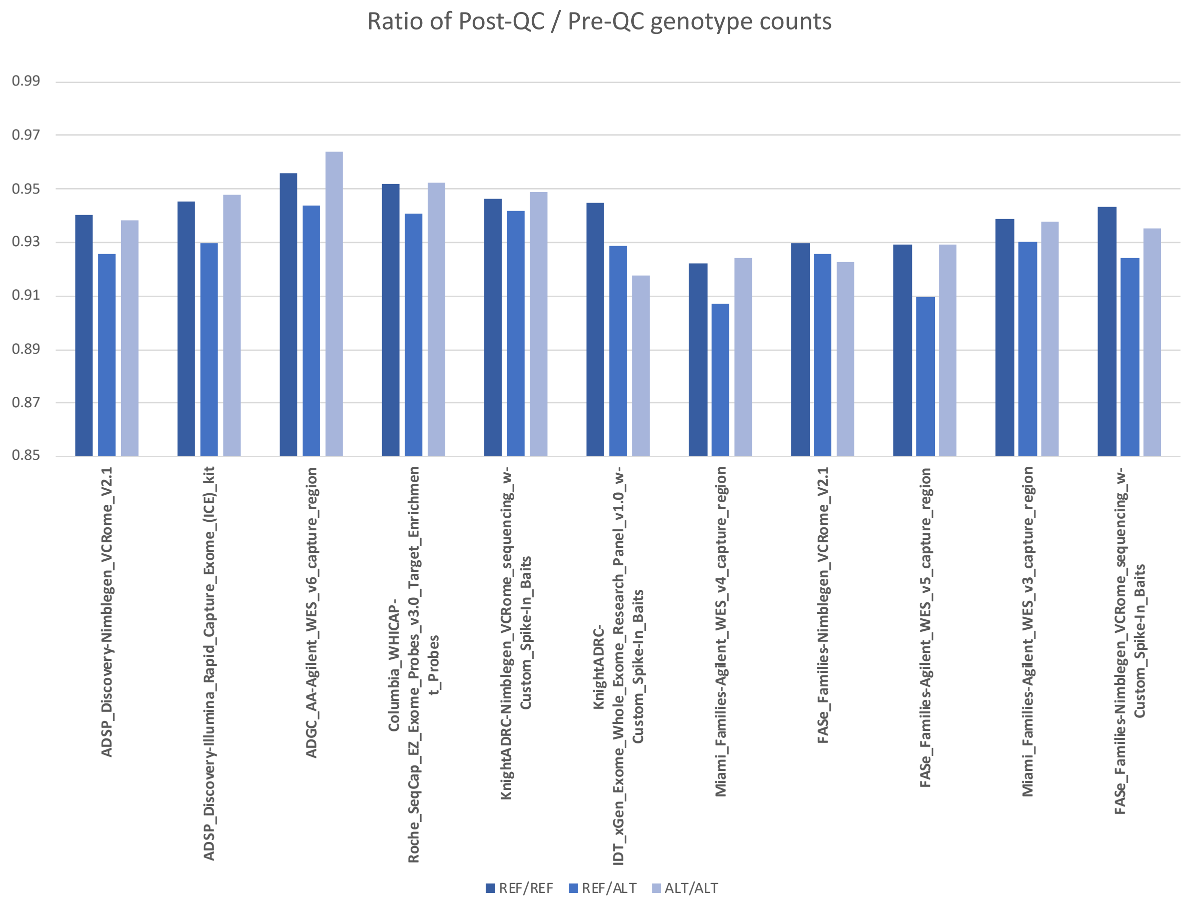

Supplementary Fig. 4 – Percentage of variants by deciles of call rate for each QC subset, prior to QC (in blue) and after (in orange).

a. AA-Agilent_WES_v6_capture_region. N = 3157 samples.

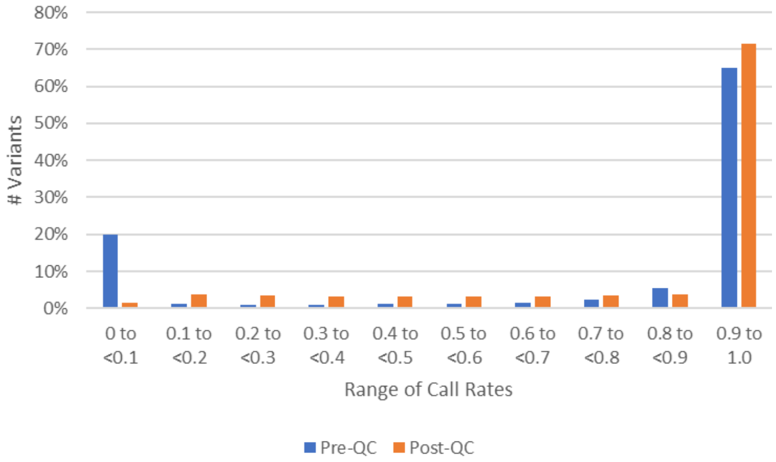

b. ADSP_Discovery-Illumina_Rapid_Capture_Exome_ICE_kit. N = 4585 samples.

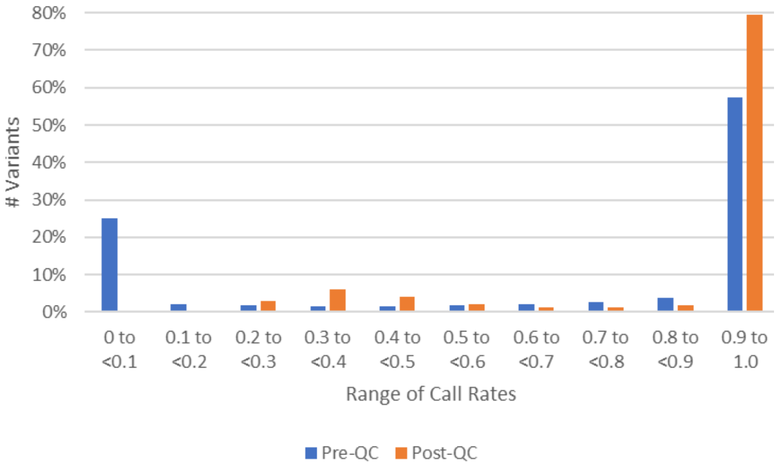

c. ADSP_Discovery-Nimblegen_VCRome_V2.1. N = 6072 samples.

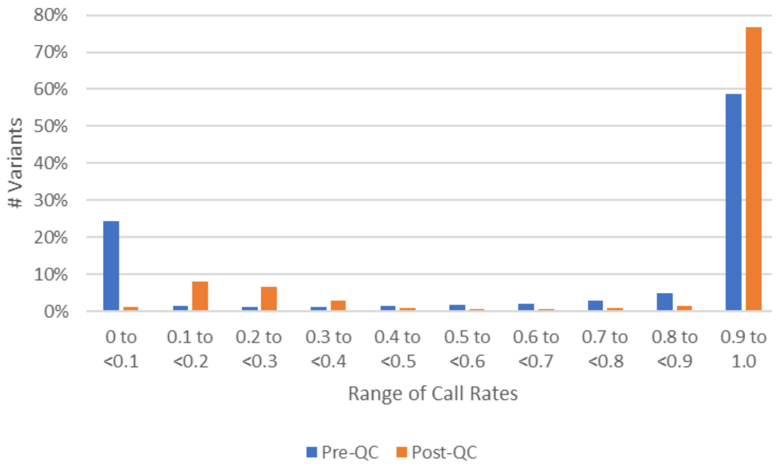

d. Brkanac_Families-Roche_SeqCap_EZ_Exome_Probes_v2.0_Target_Enrichment_Probes. N = 75 samples.

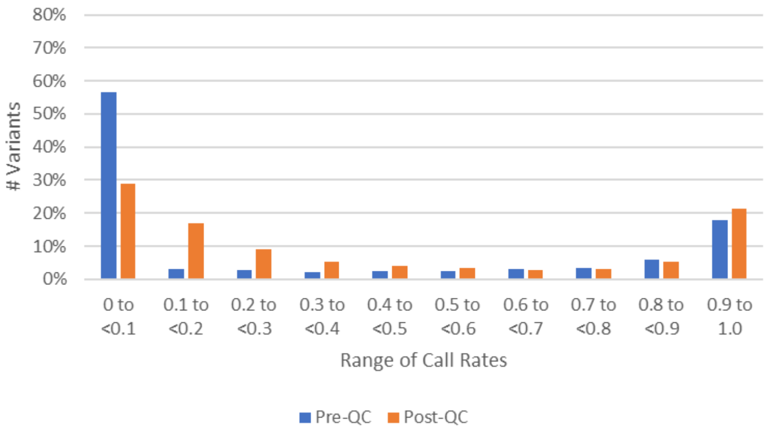

e. CBD-Agilent_WES_v5_capture_region. N = 346 samples.

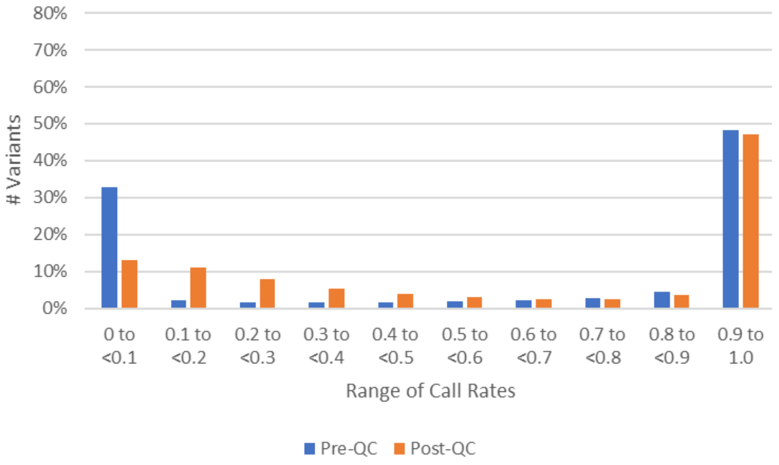

f. Columbia_WHICAP-Roche_SeqCap_EZ_Exome_Probes_v3.0_Target_Enrichment_Probes. N = 3861 samples.

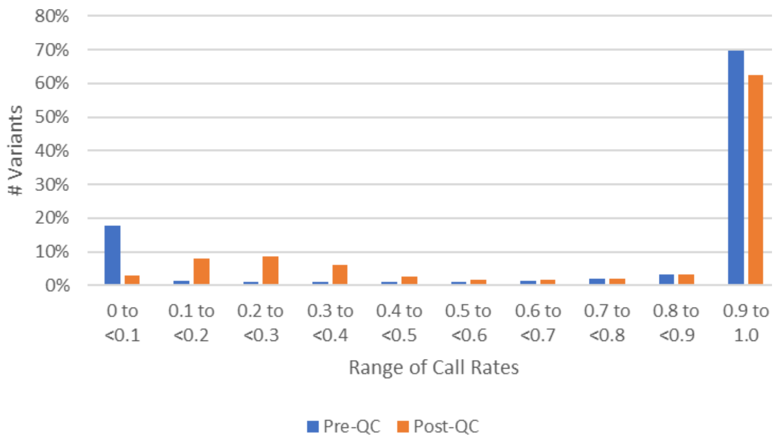

g. FASe_Families-Agilent_WES_v5_capture_region. N = 714 samples.

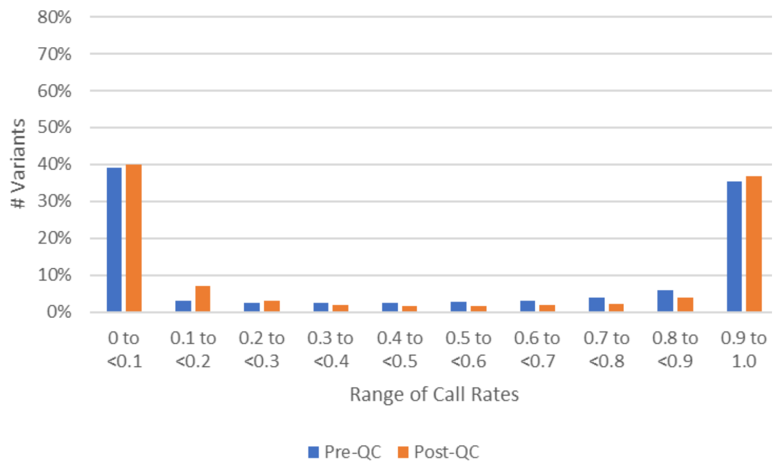

h. FASe_Families-Nimblegen_VCRome_sequencing_w-Custom_Spike-In_Baits. N = 164 samples.

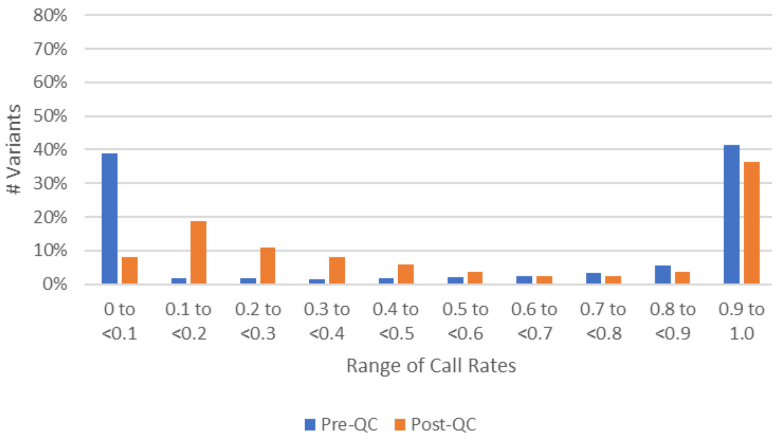

i. FASe_Families-Nimblegen_VCRome_V2. N = 222 samples.

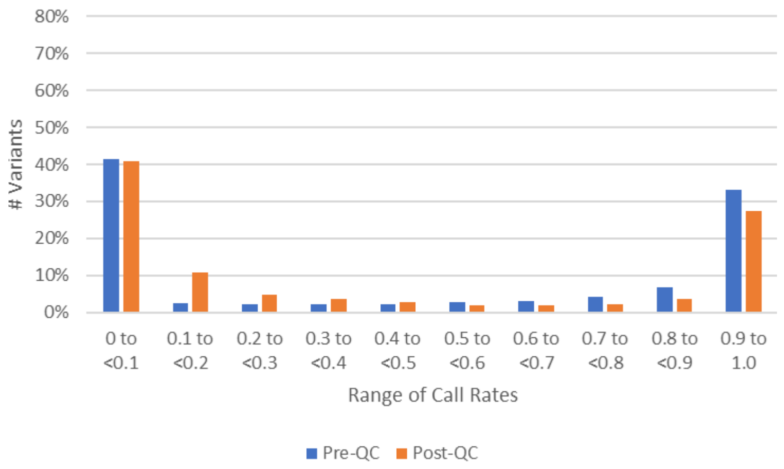

j. KnightADRC-IDT_xGen_Exome_Whole_Exome_Research_Panel_v1.0_w-Custom_Spike-In_Baits. N = 72 samples.

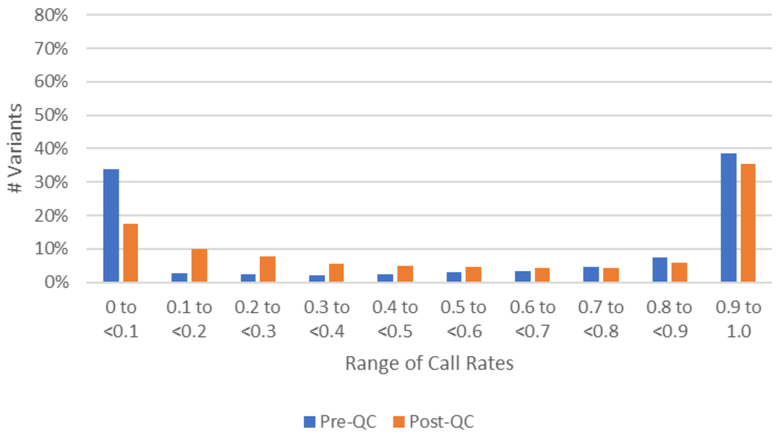

k. KnightADRC-Nimblegen_VCRome_sequencing_w-Custom_Spike-In_Baits. N = 578 samples.

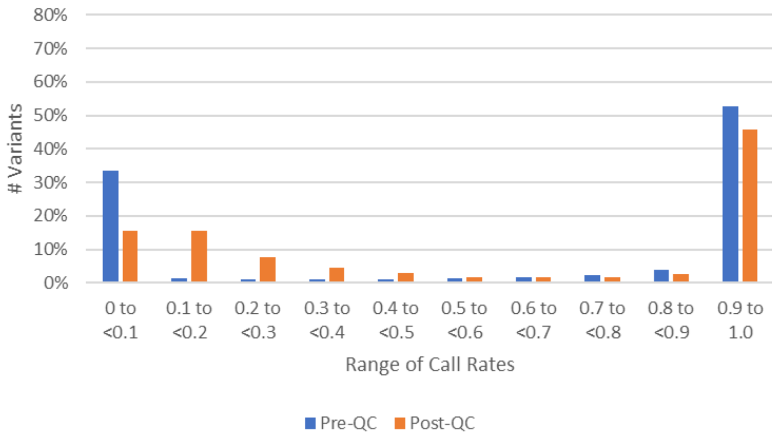

l. Miami_Families-Agilent_WES_v3_capture_region. N = 61 samples.

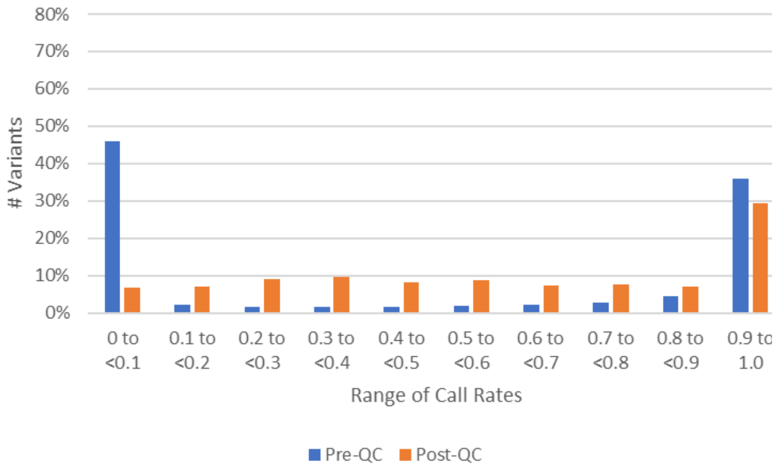

m. Miami_Families-Agilent_WES_v4_capture_region. N = 47 samples.

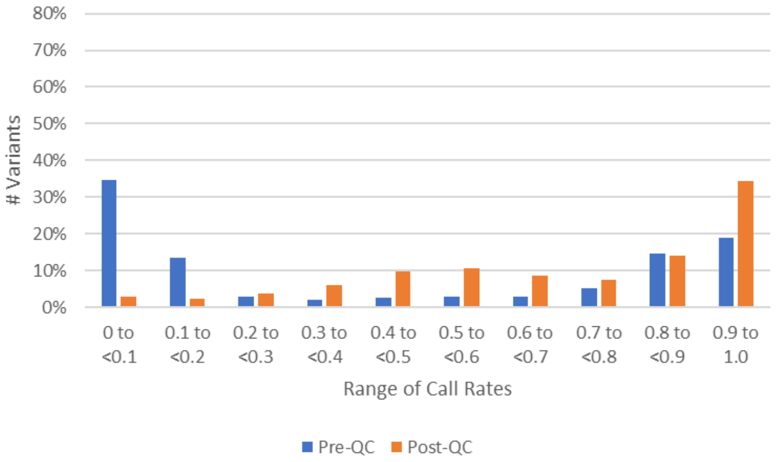

n. PSP_Nimblegen_VCRomev2.1. N = 550 samples.

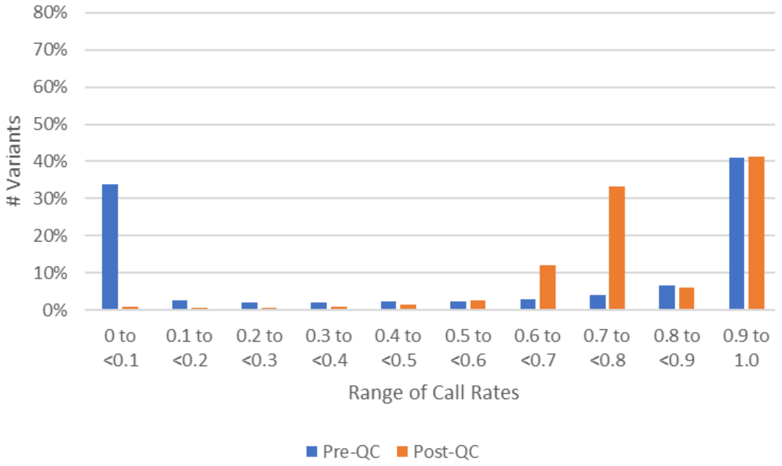

Supplementary Fig. 5 – Annotation results for all variants called in the WES pVCF of 20,504 samples: **a** Top ten categories of VEP predicted consequence; **b** Distribution of CADD phred-normalized scores.

a

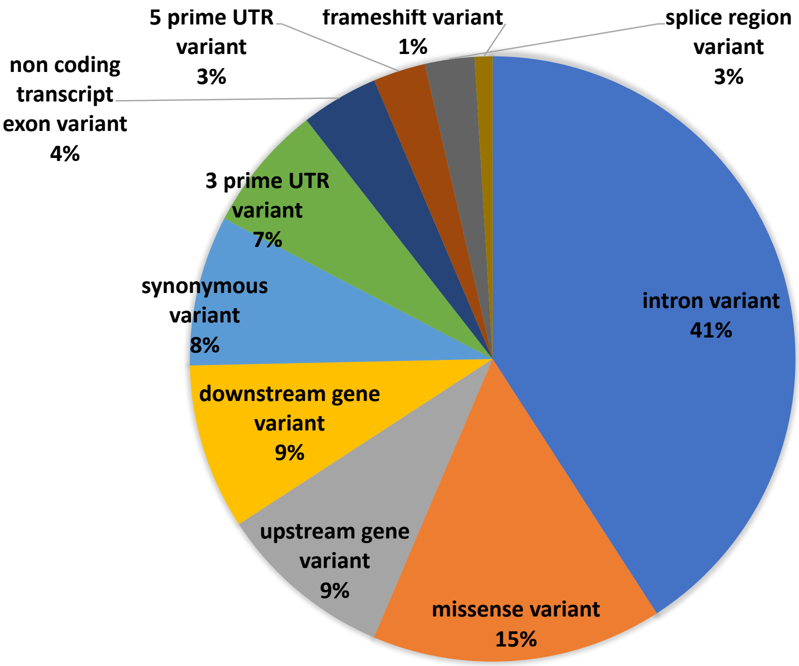

b

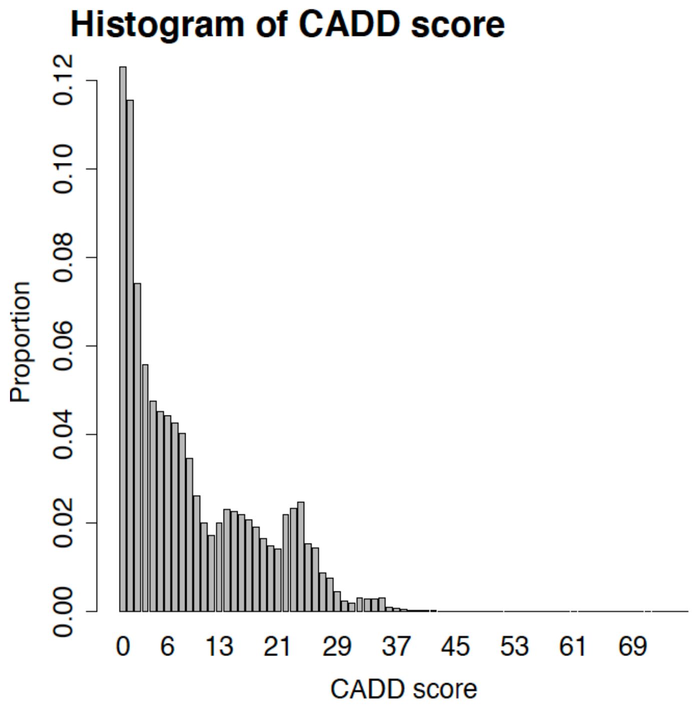

SUPPLEMENTARY TABLES

Supplementary Table 1 – Comparison of mapping percentage across sequencing centers. Wilcoxon signed rank tests were performed between every two sequencing centers and *p*-values are shown. Statistically significant results (Bonferroni corrected *p*<0.05) are highlighted.

	Baylor	Broad	CHOP	CU_IGM	FGC	IDOM	MGI	Otogenetics	PGFI	UM_HIHG	UW Genome Sciences	WashU
**Baylor**	NA											
**Broad**	0.009	NA										
**CHOP**	0.001	0.000	NA									
**CU_IGM**	0.061	0.003	0.430	NA								
**FGC**	0.949	0.010	0.001	0.047	NA							
**IDOM**	0.308	0.082	0.001	0.028	0.401	NA						
**MGI**	0.014	0.001	0.293	1.000	0.011	0.004	NA					
**Otogenetics**	0.011	0.000	0.094	0.718	0.018	0.002	0.532	NA				
**PGFI**	0.490	0.151	0.008	0.076	0.519	0.949	0.040	0.021	NA			
**UM_HIHG**	0.004	0.001	0.324	0.212	0.003	0.003	0.131	0.107	0.020	NA		
**UW Genome Sciences**	0.001	0.699	0.000	0.002	0.003	0.040	0.001	0.000	0.116	0.001	NA	
**WashU**	0.002	0.001	0.080	0.099	0.002	0.002	0.045	0.027	0.009	0.574	0.001	NA

Supplementary Table 2 – Comparison of duplicated reads percentage across sequencing centers. Wilcoxon signed rank tests were performed between every two sequencing centers and *p*-values are shown. Statistically significant results (Bonferroni corrected *p*<0.05) are highlighted.

	Baylor	Broad	CHOP	CU_IGM	FGC	IDOM	MGI	Otogenetics	PGFI	UM_HIHG	UW Genome Sciences	WashU
**Baylor**	NA											
**Broad**	0.0006	NA										
**CHOP**	0.0400	0.0400	NA									
**CU_IGM**	0.3316	0.0008	0.0759	NA								
**FGC**	0.0032	0.8470	0.0879	0.0083	NA							
**IDOM**	0.0024	0.8977	0.0473	0.0052	1.0000	NA						
**MGI**	0.0010	0.0281	0.2169	0.0032	0.2169	0.0759	NA					
**Otogenetics**	0.0400	0.0014	0.8977	0.0473	0.0158	0.0024	0.0192	NA				
**PGFI**	0.0158	0.2426	0.3653	0.0336	0.3000	0.1932	1.0000	0.1932	NA			
**UM_HIHG**	0.0024	0.6522	0.0879	0.0066	0.7969	0.6063	0.3316	0.0083	0.4779	NA		
**UW Genome Sciences**	0.6522	0.0006	0.0400	0.5190	0.0066	0.0052	0.0019	0.0233	0.0158	0.0041	NA	
**WashU**	0.0083	0.0233	0.5619	0.0281	0.1164	0.0652	0.4779	0.2169	0.6522	0.1330	0.0083	NA

Supplementary Table 3 – Comparison of paired reads percentage across sequencing centers. Wilcoxon signed rank tests were performed between every two sequencing centers and *p*-values are shown. Statistically significant results (Bonferroni corrected *p*<0.05) are highlighted.

	Baylor	Broad	CHOP	CU_IGM	FGC	IDOM	MGI	Otogenetics	PGFI	UM_HIHG	UW Genome Sciences	WashU
Baylor	NA											
Broad	0.0014	NA										
CHOP	0.2426	0.0336	NA									
CU_IGM	0.8470	0.0010	0.2703	NA								
FGC	0.2703	0.0104	0.9215	0.3244	NA							
IDOM	0.0473	0.0652	0.4385	0.0336	0.3000	NA						
MGI	0.1932	0.0024	0.7969	0.2426	0.7969	0.1330	NA					
Otogenetics	0.0041	0.0000	0.0010	0.0024	0.0019	0.0001	0.0014	NA				
PGFI	0.6063	0.0032	0.4779	0.6522	0.5619	0.1164	0.6994	0.0192	NA			
UM_HIHG	0.0066	0.4779	0.0473	0.0083	0.0281	0.1014	0.0128	0.0004	0.0158	NA		
UW Genome Sciences	0.5766	0.0000	0.3000	0.6994	0.2703	0.0192	0.2169	0.0004	0.7676	0.0010	NA	
WashU	0.7179	0.0008	0.1394	0.4779	0.1394	0.0192	0.0939	0.0095	0.4385	0.0066	0.2426	NA

Supplementary Table 4 – Comparison of quality of reads (Q30) across sequencing centers. Wilcoxon signed rank tests were performed between every two sequencing centers and *p*-values are shown. Statistically significant results (Bonferroni corrected *p*<0.05) are highlighted.

	Baylor	Broad	CHOP	CU_IGM	FGC	IDOM	MGI	Otogenetics	PGFI	UM_HIHG	UW Genome Sciences	WashU
Baylor	NA											
Broad	0.0032	NA										
CHOP	0.0000	0.0000	NA									
CU_IGM	0.0336	0.0114	0.0000	NA								
FGC	0.0006	0.0000	0.0000	0.0004	NA							
IDOM	0.0006	0.0001	0.0000	0.0004	0.6694	NA						
MGI	0.0032	0.0000	0.0000	0.0000	0.0010	0.0010	NA					
Otogenetics	0.0000	0.0000	0.0000	0.0000	0.0000	0.0000	0.0000	NA				
PGFI	0.0004	0.0004	0.0000	0.0006	0.4779	0.8470	0.0006	0.0000	NA			
UM_HIHG	0.0004	0.0004	0.0006	0.0006	0.0527	0.0336	0.0006	0.0006	0.1014	NA		
UW Genome Sciences	0.0006	0.0000	0.0000	0.0000	0.0006	0.0006	0.0104	0.0000	0.0006	0.0006	NA	
WashU	0.6994	0.0024	0.0000	0.0041	0.0004	0.0004	0.0004	0.0000	0.0004	0.0004	0.0002	NA

Supplementary Table 5 – Comparison of mapping percentage across sequencing platforms. Wilcoxon signed rank tests were performed between every two sequencing platforms and *p*-values are shown. Statistically significant results (Bonferroni corrected *p*<0.05) are highlighted.

	HiSeq2000/2500	HiSeq3000	HiSeq4000
HiSeq2000/2500	NA		
HiSeq3000	0.5326	NA	
HiSeq4000	0.1481	0.0043	NA

Supplementary Table 6 – Comparison of duplicated reads percentage across sequencing platforms. Wilcoxon signed rank tests were performed between every two sequencing platforms and *p*-values are shown. Statistically significant results (Bonferroni corrected *p*<0.05) are highlighted.

	HiSeq2000/2500	HiSeq3000	HiSeq4000
HiSeq2000/2500	NA		
HiSeq3000	0.2119	NA	
HiSeq4000	1.0000	0.1513	NA

Supplementary Table 7 – Comparison of paired reads percentage across sequencing platforms. Wilcoxon signed rank tests were performed between every two sequencing platforms and *p*-values are shown. Statistically significant results (Bonferroni corrected *p*<0.05) are highlighted.

	HiSeq2000/2500	HiSeq3000	HiSeq4000
HiSeq2000/2500	NA		
HiSeq3000	0.8437	NA	
HiSeq4000	0.6994	0.8470	NA

Supplementary Table 8 – Comparison of quality of reads (Q30) across sequencing platforms. Wilcoxon signed rank tests were performed between every two sequencing platforms and *p*-values are shown. Statistically significant results (Bonferroni corrected *p*<0.05) are highlighted.

	HiSeq2000/2500	HiSeq3000	HiSeq4000
HiSeq2000/2500	NA		
HiSeq3000	0.0083	NA	
HiSeq4000	0.0006	0.0006	NA

Supplementary Table 9 – Comparison of 20x coverage across sequencing centers. Wilcoxon signed rank tests were performed between every two sequencing centers and *p*-values are shown. Statistically significant results (Bonferroni corrected *p*<0.05) are highlighted.

	Baylor	Broad	CHOP	CU_IGM	FGC	IDOM	MGI	Otogenetics	PGFI	UM_HIHG	UW Genome Sciences	WashU
**Baylor**	NA											
**Broad**	0.0000	NA										
**CHOP**	0.0000	0.0000	NA									
**CU_IGM**	0.0000	0.0000	0.0000	NA								
**FGC**	0.0006	0.0000	0.0004	0.0003	NA							
**IDOM**	0.0003	0.0001	0.0006	0.0004	0.0000	NA						
**MGI**	0.0000	0.0000	0.0000	0.0000	0.0000	0.0000	NA					
**Otogenetics**	0.0000	0.0000	0.0000	0.0000	0.0000	0.0000	0.0000	NA				
**PGFI**	0.0004	0.0003	0.0004	0.0004	0.0000	0.0000	0.0004	0.0000	NA			
**UM_HIHG**	0.0001	0.0001	0.0001	0.0001	0.0001	0.0001	0.0001	0.0001	0.0001	NA		
**UW Genome Sciences**	0.0000	0.0000	0.0000	0.0000	0.0000	0.0000	0.0000	0.0000	0.0000	0.0000	NA	
**WashU**	0.0001	0.0001	0.0001	0.0001	0.0001	0.0001	0.0001	0.0001	0.0001	0.0001	0.0001	NA

Supplementary Table 10 – Comparison of 20x coverage across sequencing platforms. Wilcoxon signed rank tests were performed between every two sequencing platforms and *p*-values are shown. Statistically significant results (Bonferroni corrected *p*<0.05) are highlighted.

	HiSeq2000/2500	HiSeq3000	HiSeq4000
**HiSeq2000/2500**	NA		
**HiSeq3000**	0.0064	NA	
**HiSeq4000**	0.0567	0.0006	NA

Supplementary Table 11 – Allele counts over all synonymous variants within QC subsets among cases and among controls, with average ALT allele frequencies over all synonymous variants also reported separately for cases and controls for the 4 QC subsets from the three biggest studies: ADSP_Discovery, ADGC_AA and Columbia_WHICAP.

QC Subset	Total Allele Counts (Synonymous Variants)		Total ALT Allele Counts (Synonymous Variants)		Average ALT Allele Frequencies (Synonymous Variants)	
	Cases	Controls	Cases	Controls	Cases	Controls
ADSP_Discovery_Nimblegen_VCRome_V2.1	2,068,359,544	2,080,782,920	41,850,516	43,081,082	0.0202	0.0207
ADSP_Discovery_Illumina_Rapid_Capture_Exome_(ICE)_kit	1,431,099,876	818,836,140	20,555,712	12,340,924	0.0144	0.0151
ADGC_AA_Agilent_WES_v6_capture_region	718,250,500	918,464,848	33,360,892	42,577,666	0.0464	0.0464
Columbia_WHICAP_Roche_SeqCap_EZ_Exome_Probes_v3.0_Target_Enrichment_Probes	477,719,866	1,768,017,874	12,994,205	48,742,591	0.0272	0.0276

Supplementary Table 12 – Genotype counts for three genotypes (REF/REF, REF/ALT, and ALT/ALT) from Pre- and Post-QC datasets for each QC subset. The ratios of Post-QC to Pre-QC genotype counts are also shown for each genotype and overall genotypes. Variants that were monomorphic or off-capture were excluded from both Pre- and Post-QC counts.

QC Subset	Pre-QC Genotype Counts			Post-QC Genotype Counts			Ratio of Post-QC:Pre-QC Genotype Counts			Post-QC:Pre-QC
	REF/REF	REF/ALT	ALT/ALT	REF/REF	REF/ALT	ALT/ALT	REF/REF	REF/ALT	ALT/ALT	All
ADSP_Discovery-Nimblegen_VCRome_V2.1	2868180630	52291880	27099695	2696363901	48408396	25430721	0.9401	0.92573	0.93841	0.93983
ADSP_Discovery-Illumina_Rapid_Capture_Exome_(ICE)_kit	2100773103	37689890	19271835	1986325319	35044373	18268442	0.94552	0.92981	0.94793	0.94527
ADGC_AA-Agilent_WES_v6_capture_region	1594580489	29644709	14152932	1524254116	27985690	13645897	0.9559	0.94404	0.96417	0.95575
Columbia_WHICAP-Roche_SeqCap_EZ_Exome_Probes_v3.0_Target_Enrichment_Probes	1058608814	20069995	10763712	1007544687	18883718	10248788	0.95176	0.94089	0.95216	0.95157
KnightADRC-Nimblegen_VCRome_sequencing_w-Custom_Spike-In_Baits	1240588910	23275323	11737319	1173723237	21917352	11135406	0.9461	0.94166	0.94872	0.94604
KnightADRC-IDT_xGen_Exome_Whole_Exome_Research_Panel_v1.0_w-Custom_Spike-In_Baits	1401985292	31020152	14808859	1324696126	28815709	13591993	0.94487	0.92894	0.91783	0.94425
Miami_Families-Agilent_WES_v4_capture_region	1415133477	28685048	12382058	1305012305	26019035	11442995	0.92218	0.90706	0.92416	0.9219
FASe_Families-Nimblegen_VCRome_V2.1	1012890591	18451199	9318351	941725853	17083327	8598407	0.92974	0.92587	0.92274	0.92961
FASe_Families-Agilent_WES_v5_capture_region	1240991969	22325551	11178264	1153227385	20312349	10386929	0.92928	0.90983	0.92921	0.92894
Miami_Families-Agilent_WES_v3_capture_region	1144316532	22494715	11658550	1074539637	20922398	10931179	0.93902	0.9301	0.93761	0.93884
FASe_Families-Nimblegen_VCRome_sequencing_w-Custom_Spike-In_Baits	1434460815	27244883	13886525	1353178000	25175789	12989006	0.94334	0.92406	0.93537	0.9429

Supplementary Table 13 – Comparison of the bioinformatics pipeline used in Holstege *et al.,* 2022 versus VCPA-WES.

**Features**	**Holstege *et al*., 2022**	**ADSP/GCAD VCPA-WES pipeline**
Genome-build	GRCh37/hg19	GRCh38/hg38
dbSNP version	v150	v138
GATK version	v3.8	V4.1.1
Ability to process data in different genome builds	No	Can handle processed data of previous genome builds, GRCh36/hg18, GRCh37/hg19
Mark duplicates	Picard	BAMUTIL dedup_lowmem
Filer read alignments of possible chimeric origin	Yes	No
GATK BQSR	On capture regions with padding	No restrictions
GATK Haplotypecaller	On capture regions with padding	No restrictions
GATK combineGVCFs	Per study by batches (max 500 samples)	Across all studies (no limit on number of samples)
GATK genotypeGVCFs	Setting max-alternate-alleles to 20	Use best practice settings as suggested by GATK

SUPPLEMENTARY NOTES

Complete acknowledgements

Acknowledgment statement for any data distributed by NIAGADS:
Data for this study were prepared, archived, and distributed by the National Institute on Aging Alzheimer's Disease Data Storage Site (NIAGADS) at the University of Pennsylvania (U24-AG041689), funded by the National Institute on Aging.

ADSP Discovery + Extension only
The Alzheimer's Disease Sequencing Project (ADSP) is comprised of two Alzheimer's Disease (AD) genetics consortia and three National Human Genome Research Institute (NHGRI) funded Large Scale Sequencing and Analysis Centers (LSAC). The two AD genetics consortia are the Alzheimer's Disease Genetics Consortium (ADGC) funded by NIA (U01 AG032984), and the Cohorts for Heart and Aging Research in Genomic Epidemiology (CHARGE) funded by NIA (R01 AG033193), the National Heart, Lung, and Blood Institute (NHLBI), other National Institute of Health (NIH) institutes and other foreign governmental and non-governmental organizations. The Discovery Phase analysis of sequence data is supported through UF1AG047133 (to Drs. Schellenberg, Farrer, Pericak-Vance, Mayeux, and Haines); U01AG049505 to Dr. Seshadri; U01AG049506 to Dr. Boerwinkle; U01AG049507 to Dr. Wijsman; and U01AG049508 to Dr. Goate and the Discovery Extension Phase analysis is supported through U01AG052411 to Dr. Goate, U01AG052410 to Dr. Pericak-Vance and U01 AG052409 to Drs. Seshadri and Fornage. Data generation and harmonization in the Follow-up Phases is supported by U54AG052427 (to Drs. Schellenberg and Wang).

The ADGC cohorts include: Adult Changes in Thought (ACT), the Alzheimer's Disease Centers (ADC), the Chicago Health and Aging Project (CHAP), the Memory and Aging Project (MAP), Mayo Clinic (MAYO), Mayo Parkinson's Disease controls, University of Miami, the Multi-Institutional Research in Alzheimer's Genetic Epidemiology Study (MIRAGE), the National Cell Repository for Alzheimer's Disease (NCRAD), the National Institute on Aging Late Onset Alzheimer's Disease Family Study (NIA-LOAD), the Religious Orders Study (ROS), the Texas Alzheimer's Research and Care Consortium (TARC), Vanderbilt University/Case Western Reserve University (VAN/CWRU), the Washington Heights-Inwood Columbia Aging Project (WHICAP) and the Washington University Sequencing Project (WUSP), the Columbia University Hispanic- Estudio Familiar de Influencia Genetica de Alzheimer (EFIGA), the University of Toronto (UT), and Genetic Differences (GD).

The CHARGE cohorts are supported in part by National Heart, Lung, and Blood Institute (NHLBI) infrastructure grant HL105756 (Psaty), RC2HL102419 (Boerwinkle) and the neurology working group is supported by the National Institute on Aging (NIA) R01 grant AG033193. The CHARGE cohorts participating in the ADSP include the following: Austrian Stroke Prevention Study (ASPS), ASPS-Family study, and the Prospective Dementia Registry-Austria (ASPS/PRODEM-Aus), the Atherosclerosis Risk in Communities (ARIC) Study, the Cardiovascular Health Study (CHS), the Erasmus

Rucphen Family Study (ERF), the Framingham Heart Study (FHS), and the Rotterdam Study (RS). ASPS is funded by the Austrian Science Fond (FWF) grant number P20545-P05 and P13180 and the Medical University of Graz. The ASPS-Fam is funded by the Austrian Science Fund (FWF) project I904),the EU Joint Programme - Neurodegenerative Disease Research (JPND) in frame of the BRIDGET project (Austria, Ministry of Science) and the Medical University of Graz and the Steiermärkische Krankenanstalten Gesellschaft. PRODEM-Austria is supported by the Austrian Research Promotion agency (FFG) (Project No. 827462) and by the Austrian National Bank (Anniversary Fund, project 15435. ARIC research is carried out as a collaborative study supported by NHLBI contracts (HHSN268201100005C, HHSN268201100006C, HHSN268201100007C, HHSN268201100008C, HHSN268201100009C, HHSN268201100010C, HHSN268201100011C, and HHSN268201100012C). Neurocognitive data in ARIC is collected by U01 2U01HL096812, 2U01HL096814, 2U01HL096899, 2U01HL096902, 2U01HL096917 from the NIH (NHLBI, NINDS, NIA and NIDCD), and with previous brain MRI examinations funded by R01-HL70825 from the NHLBI. CHS research was supported by contracts HHSN268201200036C, HHSN268200800007C, N01HC55222, N01HC85079, N01HC85080, N01HC85081, N01HC85082, N01HC85083, N01HC85086, and grants U01HL080295 and U01HL130114 from the NHLBI with additional contribution from the National Institute of Neurological Disorders and Stroke (NINDS). Additional support was provided by R01AG023629, R01AG15928, and R01AG20098 from the NIA. FHS research is supported by NHLBI contracts N01-HC-25195 and HHSN268201500001I. This study was also supported by additional grants from the NIA (R01s AG054076, AG049607 and AG033040 and NINDS (R01 NS017950). The ERF study as a part of EUROSPAN (European Special Populations Research Network) was supported by European Commission FP6 STRP grant number 018947 (LSHG-CT-2006-01947) and also received funding from the European Community's Seventh Framework Programme (FP7/2007-2013)/grant agreement HEALTH-F4-2007-201413 by the European Commission under the programme "Quality of Life and Management of the Living Resources" of 5th Framework Programme (no. QLG2-CT-2002-01254). High-throughput analysis of the ERF data was supported by a joint grant from the Netherlands Organization for Scientific Research and the Russian Foundation for Basic Research (NWO-RFBR 047.017.043). The Rotterdam Study is funded by Erasmus Medical Center and Erasmus University, Rotterdam, the Netherlands Organization for Health Research and Development (ZonMw), the Research Institute for Diseases in the Elderly (RIDE), the Ministry of Education, Culture and Science, the Ministry for Health, Welfare and Sports, the European Commission (DG XII), and the municipality of Rotterdam. Genetic data sets are also supported by the Netherlands Organization of Scientific Research NWO Investments (175.010.2005.011, 911-03-012), the Genetic Laboratory of the Department of Internal Medicine, Erasmus MC, the Research Institute for Diseases in the Elderly (014-93-015; RIDE2), and the Netherlands Genomics Initiative (NGI)/Netherlands Organization for Scientific Research (NWO) Netherlands Consortium for Healthy Aging (NCHA), project 050-060-810. All studies are grateful to their participants, faculty and staff. The content of these manuscripts is solely the responsibility of the authors and does not necessarily

represent the official views of the National Institutes of Health or the U.S. Department of Health and Human Services.

The four LSACs are: the Human Genome Sequencing Center at the Baylor College of Medicine (U54 HG003273), the Broad Institute Genome Center (U54HG003067), The American Genome Center at the Uniformed Services University of the Health Sciences (U01AG057659), and the Washington University Genome Institute (U54HG003079).

Biological samples and associated phenotypic data used in primary data analyses were stored at Study Investigators institutions, and at the National Cell Repository for Alzheimer’s Disease (NCRAD, U24AG021886) at Indiana University funded by NIA. Associated Phenotypic Data used in primary and secondary data analyses were provided by Study Investigators, the NIA funded Alzheimer’s Disease Centers (ADCs), and the National Alzheimer’s Coordinating Center (NACC, U01AG016976) and the National Institute on Aging Genetics of Alzheimer’s Disease Data Storage Site (NIAGADS, U24AG041689) at the University of Pennsylvania, funded by NIA, and at the Database for Genotypes and Phenotypes (dbGaP) funded by NIH. This research was supported in part by the Intramural Research Program of the National Institutes of health, National Library of Medicine. Contributors to the Genetic Analysis Data included Study Investigators on projects that were individually funded by NIA, and other NIH institutes, and by private U.S. organizations, or foreign governmental or nongovernmental organizations.

For investigators using ADGC data:
The Alzheimer’s Disease Genetics Consortium (ADGC) supported sample preparation, sequencing and data processing through NIA grant U01AG032984. Sequencing data generation and harmonization is supported by the Genome Center for Alzheimer’s Disease, U54AG052427, and data sharing is supported by NIAGADS, U24AG041689. Samples from the National Centralized Repository for Alzheimer’s Disease and Related Dementias (NCRAD), which receives government support under a cooperative agreement grant (U24 AG021886) awarded by the National Institute on Aging (NIA), were used in this study. We thank contributors who collected samples used in this study, as well as patients and their families, whose help and participation made this work possible.

For use with the ADGC_AA_WES (snd10003) data:
NIH grants supported enrollment and data collection for the individual studies including: GenerAAtions R01AG20688 (PI M. Daniele Fallin, PhD); Miami/Duke R01 AG027944, R01 AG028786 (PI Margaret A. Pericak-Vance, PhD); NC A&T P20 MD000546, R01 AG28786-01A1 (PI Goldie S. Byrd, PhD); Case Western (PI Jonathan L. Haines, PhD); MIRAGE R01 AG009029 (PI Lindsay A. Farrer, PhD); ROS P30AG10161, R01AG15819, R01AG30146, TGen (PI David A. Bennett, MD); MAP R01AG17917, R01AG15819, TGen (PI David A. Bennett, MD); MARS R01AG022018 (PI Lisa L. Barnes).[CL1] [KA2] The NACC database is funded by NIA/NIH Grant U01 AG016976. NACC data are contributed by the NIA-funded ADCs: P30 AG019610 (PI Eric Reiman, MD), P30 AG013846 (PI Neil Kowall, MD), P30 AG062428-01 (PI James Leverenz, MD)

P50 AG008702 (PI Scott Small, MD), P50 AG025688 (PI Allan Levey, MD, PhD), P50 AG047266 (PI Todd Golde, MD, PhD), P30 AG010133 (PI Andrew Saykin, PsyD), P50 AG005146 (PI Marilyn Albert, PhD), P30 AG062421-01 (PI Bradley Hyman, MD, PhD), P30 AG062422-01 (PI Ronald Petersen, MD, PhD), P50 AG005138 (PI Mary Sano, PhD), P30 AG008051 (PI Thomas Wisniewski, MD), P30 AG013854 (PI Robert Vassar, PhD), P30 AG008017 (PI Jeffrey Kaye, MD), P30 AG010161 (PI David Bennett, MD), P50 AG047366 (PI Victor Henderson, MD, MS), P30 AG010129 (PI Charles DeCarli, MD), P50 AG016573 (PI Frank LaFerla, PhD), P30 AG062429-01(PI James Brewer, MD, PhD), P50 AG023501 (PI Bruce Miller, MD), P30 AG035982 (PI Russell Swerdlow, MD), P30 AG028383 (PI Linda Van Eldik, PhD), P30 AG053760 (PI Henry Paulson, MD, PhD), P30 AG010124 (PI John Trojanowski, MD, PhD), P50 AG005133 (PI Oscar Lopez, MD), P50 AG005142 (PI Helena Chui, MD), P30 AG012300 (PI Roger Rosenberg, MD), P30 AG049638 (PI Suzanne Craft, PhD), P50 AG005136 (PI Thomas Grabowski, MD), P30 AG062715-01 (PI Sanjay Asthana, MD, FRCP), P50 AG005681 (PI John Morris, MD), P50 AG047270 (PI Stephen Strittmatter, MD, PhD).

For investigators using WHICAP data:
Data collection and sharing for this project was supported by the Washington Heights-Inwood Columbia Aging Project (WHICAP, PO1AG07232, R01AG037212, RF1AG054023) funded by the National Institute on Aging (NIA) and by the National Center for Advancing Translational Sciences, National Institutes of Health, through Grant Number UL1TR001873. This manuscript has been reviewed by WHICAP investigators for scientific content and consistency of data interpretation with previous WHICAP Study publications. We acknowledge the WHICAP study participants and the WHICAP research and support staff for their contributions to this study.

For investigators using HIHG_Miami_Families data:
This work was supported by the National Institutes of Health (R01 AG027944, R01 AG028786 to MAPV, R01 AG019085 to JLH, P20 MD000546); a joint grant from the Alzheimer's Association (SG-14-312644) and the Fidelity Biosciences Research Initiative to MAPV; the BrightFocus Foundation (A2011048 to MAPV). NIA-LOAD Family-Based Study supported the collection of samples used in this study through NIH grants U24 AG026395 and R01 AG041797 and the MIRAGE cohort was supported through the NIH grants R01 AG025259 and R01 AG048927. We thank contributors, including the Alzheimer's disease Centers who collected samples used in this study, as well as patients and their families, whose help and participation made this work possible. Study design: HNC, BWK, JLH, MAPV; Sample collection: MLC, JMV, RMC, LAF, JLH, MAPV; Whole exome sequencing and Sanger sequencing: SR, PLW; Sequencing data analysis: HNC, BWK, KLHN, SR, MAK, JRG, ERM, GWB, MAPV; Statistical analysis: BWK, KLHN, JMJ, MAPV; Preparation of manuscript: HNC, BWK. The authors jointly discussed the experimental results throughout the duration of the study. All authors read and approved the final manuscript.

For investigators using CBD data:
CBD Solutions funded the WES, data processing, and analysis. Assembled samples are from University College London (John Hardy), Mayo Clinic Jacksonville (Dennis

Dickson), University of Pennsylvania (John Trojanowski), Emory University (Marla Gearing), Johns Hopkins University (Alex Pantelyat), Indiana University (Bernadino Ghetti), New York Brain Bank (Jean Paul Vonsattel), McClean Brain Bank (Elaine Benes), University of Texas Southwestern (Charles White), University of California Los Angeles (William Tourtelloute), and European collaborators at University Munich and Neurobiobank Munich (Gunter Hoglinger, Ulrich Muller, Hans Kretzschmr), Newcastle University, University of Barcelona (Charles Gaig), MRC London Brain Bank, Australian Brain Bank, and the University of Madrid (Alberto Rábano Gutiérrez).

For investigators using PSP data:
This work was funded by the following NIH grants: P01 AG017586 (VM-YL, GDS, JQT), U54 NS100693 (OR, DD, GDS), UG3 NS104095 (GDS, L-SW, OR), U54 AG052427 (l-SW, GDS), P30 AG010133 (B.G.), R01 AG057516 (AC, AM, AW, JAP, SG), R01 HL143790 (AC, SG), R01 HG010067 (SG), RF1 AG055477 (CB), P01 AG017586 (VM-YL, GDS, JQT, VMV), UG3 NS104095 and CWOW grant U54 NS100693 (DD), AG025688 and NS055077 (MG), P30 AG012300 (CLW), P30 AG053760 (APL and RA), 1P50NS091856 (RA), 5 P50 AG005134 (MPF), AG005131 (DRG), Johns Hopkins University Morris K. Udall Parkinson's Disease Research Center of Excellence grant P50 NS038377 and Alzheimer's Disease Research Center grant P50 AG05146 (JCT), U24 NS072026 and P30 AG19610 (TGB). This work was also funded by Cure PSP (GDS), the Rainwater Foundation (GDS), the Daniel B. Burke Endowed Chair for Diabetes Research (SG), the CHOP Center for Spatial and Functional Genomics (AW, SFG), a CUREPSP research grant (Cure PSP Grant # 515-14; 2013-2015) to P.P., the Reta Lila Weston Trust for Medical Research, the PSP Association (RdS), and the Michael J. Fox Foundation for Parkinson's Research (TGB). G. Höglinger was funded by the German Research Foundation (DFG) under Germany's Excellence Strategy within the framework of the Munich Cluster for Systems Neurology (EXC 2145 SyNergy – ID 390857198), the German Federal Ministry of Education and Research (BMBF, 01KU1403A EpiPD; 01EK1605A HitTau), and the NOMIS foundation (FTLD project). J. Hardy was partly funded by UKDRI limited which receives its funding from the MRC, the Alzheimer's Society and Alzheimer Research UK. The London Neurodegenerative Diseases Brain Bank receives funding from the UK Medical Research Council (MR/L016397/1) and as part of the Brains for Dementia Research programme, jointly funded by Alzheimer's Research UK and the Alzheimer's Society. Queen Square Brain Bank is supported by the Reta Lila Weston Institute for Neurological Studies and the Medical Research Council UK. Newcastle Brain Tissue Resource is funded in part by a grant from the UK Medical Research Council (MR/L016451/1) and by Brains for Dementia Research, a joint venture between Alzheimer's Society and Alzheimer's Research UK (CMM) and National Institute of Health Research Biomedical Research Centre at Newcastle upon Tyne Hospitals NHS Foundation Trust and Newcastle University (CMM). This work was partly funded by UKDRI limited which receives its funding from the MRC, the Alzheimer's Society and Alzheimer Research UK (JH). The Mayo Clinic Florida had support from a Morris K. Udall Parkinson's Disease Research Center of Excellence (NINDS P50 #NS072187), CurePSP and the Tau Consortium. OAR is supported by a NINDS Tau Center without Walls (U54-NS100693), NINDS R01-NS078086 and the Mayo Clinic Center for Individualized Medicine. Funding provided by

CurePSP through the generous support of the Peebler PSP Research Foundation in memory of Charles D. Peebler Jr. and Drs. Jeffrey S. and Jennifer R. Friedman in memory of Morton L. Friedman.

For investigators using KnightADRC data:
This work was supported by grants from the National Institutes of Health (R01AG044546, P01AG003991, RF1AG053303, R01AG058501, U01AG058922, RF1AG058501 and R01AG057777). The recruitment and clinical characterization of research participants at Washington University were supported by NIH P50 AG05681, P01 AG03991, and P01 AG026276. This work was supported by access to equipment made possible by the Hope Center for Neurological Disorders, and the Departments of Neurology and Psychiatry at Washington University School of Medicine.
We thank the contributors who collected samples used in this study, as well as patients and their families, whose help and participation made this work possible. This work was supported by access to equipment made possible by the Hope Center for Neurological Disorders, and the Departments of Neurology and Psychiatry at Washington University School of Medicine

For investigators using FASe_Families data:
This work was supported by grants from the National Institutes of Health (R01AG044546, P01AG003991, RF1AG053303, R01AG058501, U01AG058922, RF1AG058501 and R01AG057777). The recruitment and clinical characterization of research participants at Washington University were supported by NIH P50 AG05681, P01 AG03991, and P01 AG026276. This work was supported by access to equipment made possible by the Hope Center for Neurological Disorders, and the Departments of Neurology and Psychiatry at Washington University School of Medicine.
We thank the contributors who collected samples used in this study, as well as patients and their families, whose help and participation made this work possible. This work was supported by access to equipment made possible by the Hope Center for Neurological Disorders, and the Departments of Neurology and Psychiatry at Washington University School of Medicine

For investigators using Brkanac_Families data:

This work was partially supported by grant funding from NIH R01 AG039700 and NIH P50 AG005136. Subjects and samples used here were originally collected with grant funding from NIH U24 AG026395, U24 AG021886, P50 AG008702, P01 AG007232, R37 AG015473, P30 AG028377, P50 AG05128, P50 AG16574, P30 AG010133, P50 AG005681, P01 AG003991, U01MH046281, U01 MH046290 and U01 MH046373. The funders had no role in study design, analysis or preparation of the manuscript. The authors declare no competing interests.

SUPPLEMENTARY REFERENCES

1) Holstege H., et al. Exome sequencing identifies rare damaging variants in ATP8B4 and ABCA1 as risk factors for Alzheimer's disease. *Nat Genet* **54(12)**:1786-1794 (2022).
