# Supplementary material for: Human whole-exome genotype data for Alzheimer’s disease
Source: Nat Commun. 2024 Jan 23;15:684. doi: 10.1038/s41467-024-44781-7 (PMC10805795; doi:10.1038/s41467-024-44781-7)
Supplement: Supplementary file 3 — Reporting Summary [file 41467_2024_44781_MOESM3_ESM.pdf]

nature portfolio

Corresponding author(s): Yuk Yee Leung, Li-San Wang

Last updated by author(s): Nov 15, 2023

Reporting Summary

Nature Portfolio wishes to improve the reproducibility of the work that we publish. This form provides structure for consistency and transparency in reporting. For further information on Nature Portfolio policies, see our Editorial Policies and the Editorial Policy Checklist.

Statistics

For all statistical analyses, confirm that the following items are present in the figure legend, table legend, main text, or Methods section.

n/a	Confirmed	
☐	☒	The exact sample size (*n*) for each experimental group/condition, given as a discrete number and unit of measurement
☐	☒	A statement on whether measurements were taken from distinct samples or whether the same sample was measured repeatedly
☐	☒	The statistical test(s) used AND whether they are one- or two-sided<br>*Only common tests should be described solely by name; describe more complex techniques in the Methods section.*
☒	☐	A description of all covariates tested
☒	☐	A description of any assumptions or corrections, such as tests of normality and adjustment for multiple comparisons
☐	☒	A full description of the statistical parameters including central tendency (e.g. means) or other basic estimates (e.g. regression coefficient) AND variation (e.g. standard deviation) or associated estimates of uncertainty (e.g. confidence intervals)
☐	☒	For null hypothesis testing, the test statistic (e.g. *F*, *t*, *r*) with confidence intervals, effect sizes, degrees of freedom and *P* value noted<br>*Give P values as exact values whenever suitable.*
☒	☐	For Bayesian analysis, information on the choice of priors and Markov chain Monte Carlo settings
☒	☐	For hierarchical and complex designs, identification of the appropriate level for tests and full reporting of outcomes
☒	☐	Estimates of effect sizes (e.g. Cohen's *d*, Pearson's *r*), indicating how they were calculated

Our web collection on statistics for biologists contains articles on many of the points above.

Software and code

Policy information about availability of computer code

Data collection	All the sequencing data described were processed using VCPA-WES (new pipeline, v1.1) which is available at https://bitbucket.org/NIAGADS/vcpa-pipeline/src/master/VCPA/. The code base used for processing WGS (VCPA-WGS) has been published in https://pubmed.ncbi.nlm.nih.gov/30351394/ but not for the WES part (VCPA-WES). In summary, individual calling of the samples was performed using codes from stage 0 up to stage 2b while joint genotyping were done using codes in stage 3. WES specific codes can be located by searching "WES" in the repository and are fully described in Figure 1.
Data analysis	The figures in this manuscript were generated using R version 3.6.1.

For manuscripts utilizing custom algorithms or software that are central to the research but not yet described in published literature, software must be made available to editors and reviewers. We strongly encourage code deposition in a community repository (e.g. GitHub). See the Nature Portfolio guidelines for submitting code & software for further information.

Data

Policy information about availability of data

All manuscripts must include a data availability statement. This statement should provide the following information, where applicable:

- Accession codes, unique identifiers, or web links for publicly available datasets
- A description of any restrictions on data availability
- For clinical datasets or third party data, please ensure that the statement adheres to our policy

Source data are provided with this paper. All CRAMs, gVCFs generated by GATK4.1.1, and QC-ed pVCFs of the abovementioned ADSP WES data set are available in the NIAGADS Data Sharing Service (DSS) (NG00067.v3), together with pedigree structures for family studies and phenotypes that were harmonized according to ADSP protocols. The WES target regions (from GRCh36, GRCh37) now lifted to GRCh38 for analyses is available at https://dss.niagads.org/wp-content/uploads/2021/08/gcad.wes_.20650.VCPA1_.1.2019.11.01.targetregions.zip?x78736. Qualified investigators can access these data with a submission request and approval from the NIAGADS Data Access Committee managed by independent NIH program officers. Data can be downloaded through the DSS portal. More information about the data set can be found on the data set page,NG00067 (https://dss.niagads.org/datasets/ng00067/). See theApplication Instructionspage (https://dss.niagads.org/documentation/applying-for-data/application-instructions/) on how to submit a Data Access Request and access data.

Research involving human participants, their data, or biological material

Policy information about studies with human participants or human data. See also policy information about sex, gender (identity/presentation), and sexual orientation and race, ethnicity and racism.

Reporting on sex and gender	Sex and gender information for all samples is described in Table 1 of the manuscript. None of the studies prioritize one sex over the other when doing recruitment nor study design. Sex and/or gender of participants was determined based on selfreport.<br>Sex check for variants outside chr X PAR region was performed to identify possible sample swaps or misreporting.
Reporting on race, ethnicity, or other socially relevant groupings	Population information related to race/ethnicity, sex and gender, age, as well as number of apoeE4 alleles can be found in Table 2 of the manuscript. Detailed population analyses results are also shown in Supplementary Figure 2.
Population characteristics	Population information related to race/ethnicity, sex and gender, age, as well as number of apoeE4 alleles can be found in Table 2 of the manuscript. Detailed population analyses results are also shown in Supplementary Figure 2.
Recruitment	All the studies information can be found in the table under https://dss.niagads.org/datasets/ng00067/. The ones with "WES" were used in this manuscript. Clicking the URL under the "Sample Set", then clicking on "Cohorts" can give you more information about the cohort.
Ethics oversight	All the studies information can be found in the table under https://dss.niagads.org/datasets/ng00067/. The ones with "WES" were used in this manuscript. Clicking the URL under the "Sample Set", then clicking on "Cohorts" can give you more information if the cohort has any ethnic protocol in place and shared with NIAGADS.

Note that full information on the approval of the study protocol must also be provided in the manuscript.

Field-specific reporting

Please select the one below that is the best fit for your research. If you are not sure, read the appropriate sections before making your selection.

☒ Life sciences ☐ Behavioural & social sciences ☐ Ecological, evolutionary & environmental sciences

For a reference copy of the document with all sections, see nature.com/documents/nr-reporting-summary-flat.pdf

Life sciences study design

All studies must disclose on these points even when the disclosure is negative.

Sample size	20504 samples were included in the output of this dataset. No sample size calculation was performed; we included everything that was possibly available at the moment when we started this project. Also, the focus of this paper is about the development of a new joint-calling pipeline for WES data and resource generation, but not on genetic data analyses.
Data exclusions	20770 samples were available and was joint called at first. QA checks were performed to identify bad quality samples. In total, we dropped 55 samples that failed the SNP concordance check, 41 samples that were recorded with the incorrect sex, and 211 samples that failed the contamination check. An additional 266 samples were dropped due to consent issues, resulting in a call set containing 20,504 samples.
Replication	No replication was performed as this is not relevant for our study, in which the focus of this paper is about the development of a new joint-calling pipeline for WES data and resource generation, but not on genetic data analyses.
Randomization	No randomization was performed as this is not relevant for our study, in which the focus of this paper is about the development of a new joint-calling pipeline for WES data and resource generation, but not on genetic data analyses.

Blinding | Blinding was not possible since phenotype variables were not used to generate the data.

Reporting for specific materials, systems and methods

We require information from authors about some types of materials, experimental systems and methods used in many studies. Here, indicate whether each material, system or method listed is relevant to your study. If you are not sure if a list item applies to your research, read the appropriate section before selecting a response.

Materials & experimental systems

n/a	Involved in the study
☒	☐ Antibodies
☒	☐ Eukaryotic cell lines
☒	☐ Palaeontology and archaeology
☒	☐ Animals and other organisms
☒	☐ Clinical data
☒	☐ Dual use research of concern
☒	☐ Plants

Methods

n/a	Involved in the study
☒	☐ ChIP-seq
☒	☐ Flow cytometry
☒	☐ MRI-based neuroimaging

Plants

Seed stocks | *Report on the source of all seed stocks or other plant material used. If applicable, state the seed stock centre and catalogue number. If plant specimens were collected from the field, describe the collection location, date and sampling procedures.*

Novel plant genotypes | *Describe the methods by which all novel plant genotypes were produced. This includes those generated by transgenic approaches, gene editing, chemical/radiation-based mutagenesis and hybridization. For transgenic lines, describe the transformation method, the number of independent lines analyzed and the generation upon which experiments were performed. For gene-edited lines, describe the editor used, the endogenous sequence targeted for editing, the targeting guide RNA sequence (if applicable) and how the editor was applied.*

Authentication | *Describe any authentication procedures for each seed stock used or novel genotype generated. Describe any experiments used to assess the effect of a mutation and, where applicable, how potential secondary effects (e.g. second site T-DNA insertions, mosiacism, off-target gene editing) were examined.*
